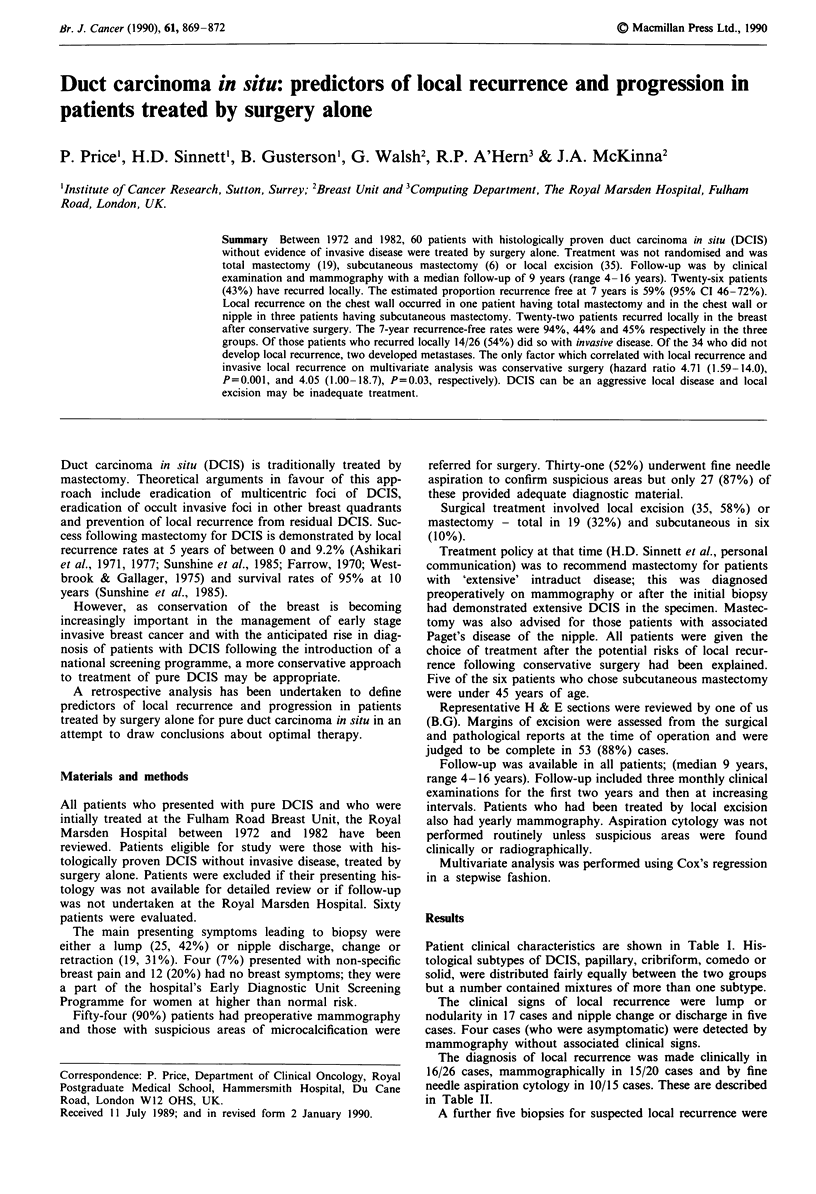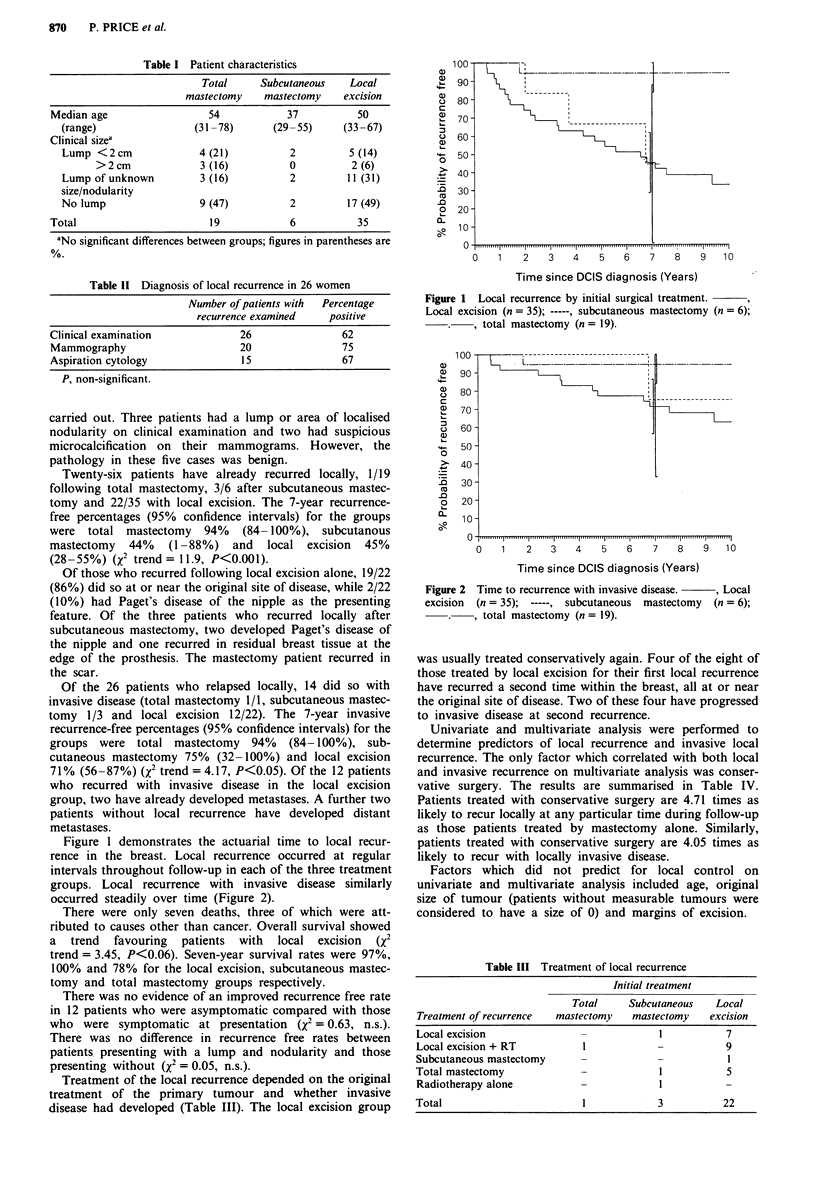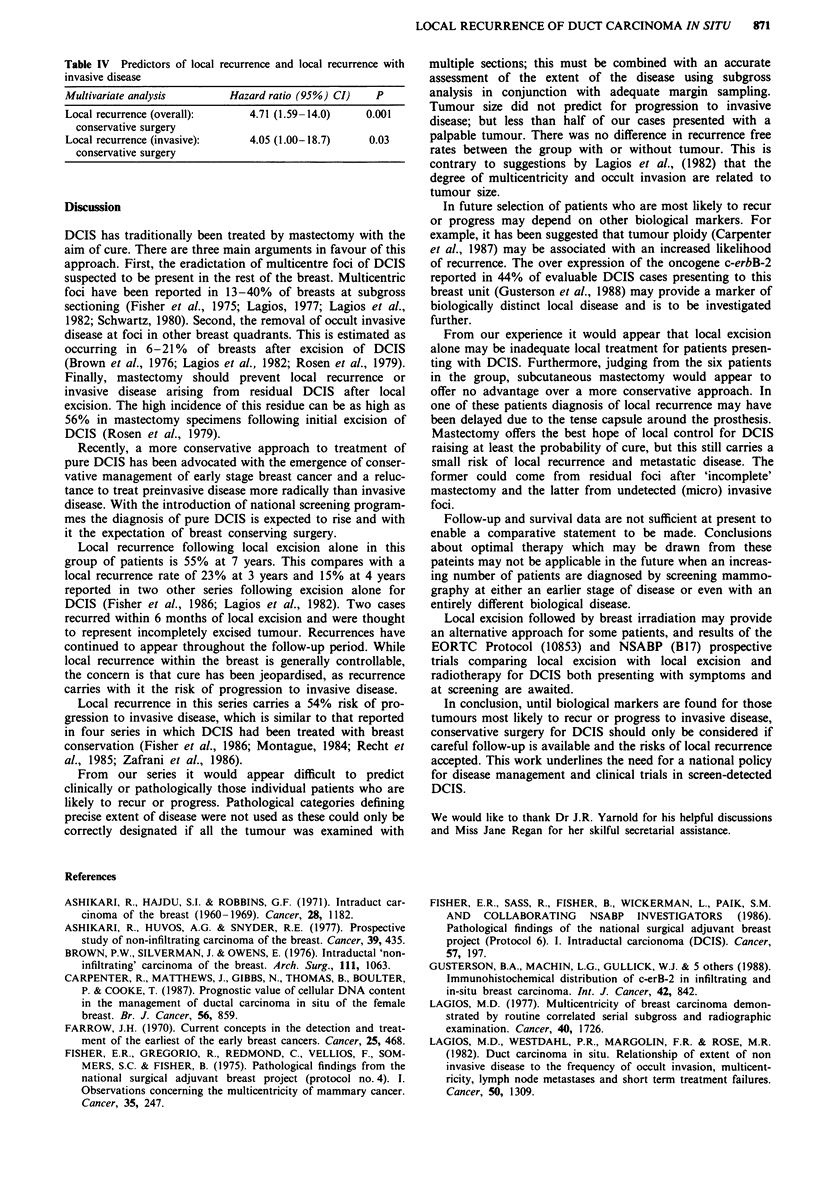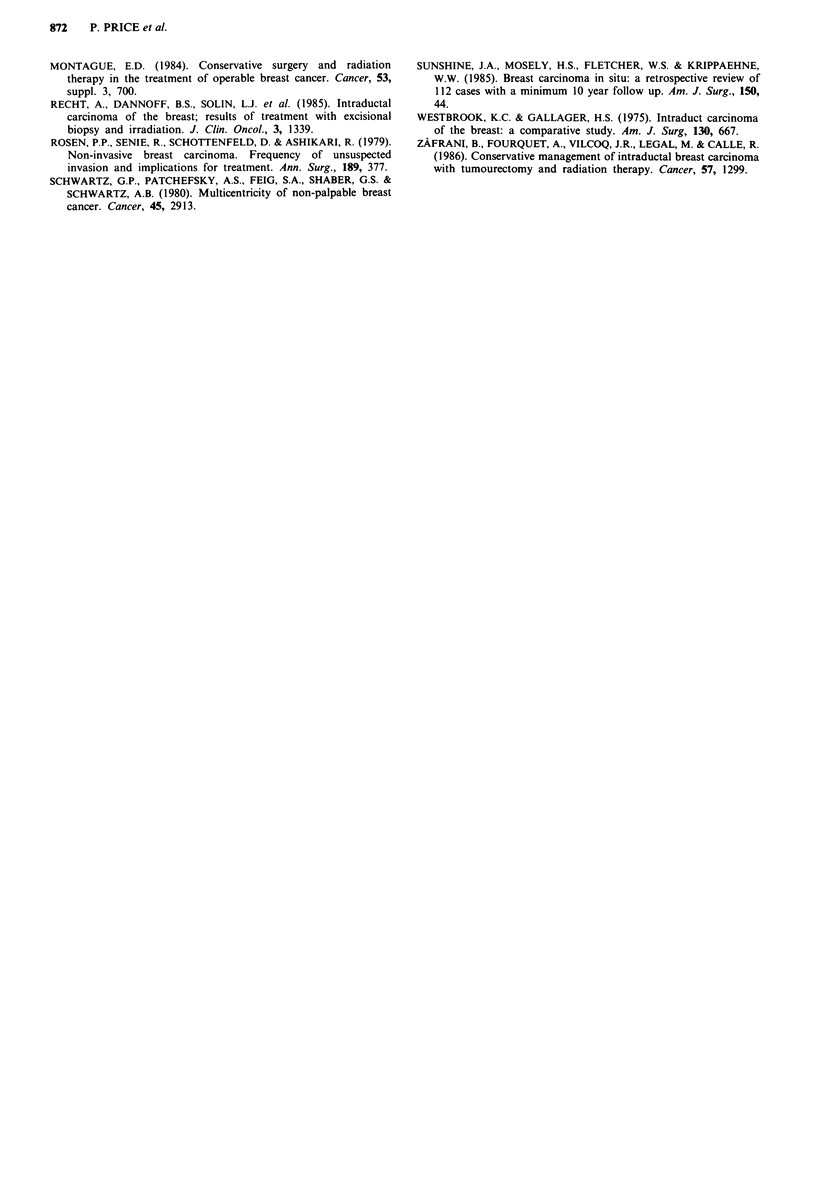# Duct carcinoma in situ: predictors of local recurrence and progression in patients treated by surgery alone.

**DOI:** 10.1038/bjc.1990.194

**Published:** 1990-06

**Authors:** P. Price, H. D. Sinnett, B. Gusterson, G. Walsh, R. P. A'Hern, J. A. McKinna

**Affiliations:** Institute of Cancer Research, Sutton, Surrey, UK.

## Abstract

Between 1972 and 1982, 60 patients with histologically proven duct carcinoma in situ (DCIS) without evidence of invasive disease were treated by surgery alone. Treatment was not randomised and was total mastectomy (19), subcutaneous mastectomy (6) or local excision (35). Follow-up was by clinical examination and mammography with a median follow-up of 9 years (range 4-16 years). Twenty-six patients (43%) have recurred locally. The estimated proportion recurrence free at 7 years is 59% (95% CI 46-72%). Local recurrence on the chest wall occurred in one patient having total mastectomy and in the chest wall or nipple in three patients having subcutaneous mastectomy. Twenty-two patients recurred locally in the breast after conservative surgery. The 7-year recurrence-free rates were 94%, 44% and 45% respectively in the three groups. Of those patients who recurred locally 14/26 (54%) did so with invasive disease. Of the 34 who did not develop local recurrence, two developed metastases. The only factor which correlated with local recurrence and invasive local recurrence on multivariate analysis was conservative surgery (hazard ratio 4.71 (1.59-14.0), P = 0.001, and 4.05 (1.00-18.7), P = 0.03, respectively). DCIS can be an aggressive local disease and local excision may be inadequate treatment.


Br. J. Cancer (1990), 61, 869-872 © Macmillan Press Ltd., 1990

### Duct carcinoma *in situ*: predictors of local recurrence and progression in patients treated by surgery alone

P. Price[1], H.D. Sinnett[1], B. Gusterson[1], G. Walsh[2], R.P. A'Hern[3] & J.A. McKinna[2]

[1]*Institute of Cancer Research, Sutton, Surrey;* [2]*Breast Unit and* [3]*Computing Department, The Royal Marsden Hospital, Fulham Road, London, UK.*

**Summary** Between 1972 and 1982, 60 patients with histologically proven duct carcinoma *in situ* (DCIS) without evidence of invasive disease were treated by surgery alone. Treatment was not randomised and was total mastectomy (19), subcutaneous mastectomy (6) or local excision (35). Follow-up was by clinical examination and mammography with a median follow-up of 9 years (range 4-16 years). Twenty-six patients (43%) have recurred locally. The estimated proportion recurrence free at 7 years is 59% (95% CI 46-72%). Local recurrence on the chest wall occurred in one patient having total mastectomy and in the chest wall or nipple in three patients having subcutaneous mastectomy. Twenty-two patients recurred locally in the breast after conservative surgery. The 7-year recurrence-free rates were 94%, 44% and 45% respectively in the three groups. Of those patients who recurred locally 14/26 (54%) did so with *invasive* disease. Of the 34 who did not develop local recurrence, two developed metastases. The only factor which correlated with local recurrence and invasive local recurrence on multivariate analysis was conservative surgery (hazard ratio 4.71 (1.59-14.0), $P=0.001$, and 4.05 (1.00-18.7), $P=0.03$, respectively). DCIS can be an aggressive local disease and local excision may be inadequate treatment.

Duct carcinoma *in situ* (DCIS) is traditionally treated by mastectomy. Theoretical arguments in favour of this approach include eradication of multicentric foci of DCIS, eradication of occult invasive foci in other breast quadrants and prevention of local recurrence from residual DCIS. Success following mastectomy for DCIS is demonstrated by local recurrence rates at 5 years of between 0 and 9.2% (Ashikari *et al.*, 1971, 1977; Sunshine *et al.*, 1985; Farrow, 1970; Westbrook & Gallager, 1975) and survival rates of 95% at 10 years (Sunshine *et al.*, 1985).

However, as conservation of the breast is becoming increasingly important in the management of early stage invasive breast cancer and with the anticipated rise in diagnosis of patients with DCIS following the introduction of a national screening programme, a more conservative approach to treatment of pure DCIS may be appropriate.

A retrospective analysis has been undertaken to define predictors of local recurrence and progression in patients treated by surgery alone for pure duct carcinoma *in situ* in an attempt to draw conclusions about optimal therapy.

### Materials and methods

All patients who presented with pure DCIS and who were intially treated at the Fulham Road Breast Unit, the Royal Marsden Hospital between 1972 and 1982 have been reviewed. Patients eligible for study were those with histologically proven DCIS without invasive disease, treated by surgery alone. Patients were excluded if their presenting histology was not available for detailed review or if follow-up was not undertaken at the Royal Marsden Hospital. Sixty patients were evaluated.

The main presenting symptoms leading to biopsy were either a lump (25, 42%) or nipple discharge, change or retraction (19, 31%). Four (7%) presented with non-specific breast pain and 12 (20%) had no breast symptoms; they were a part of the hospital's Early Diagnostic Unit Screening Programme for women at higher than normal risk.

Fifty-four (90%) patients had preoperative mammography and those with suspicious areas of microcalcification were referred for surgery. Thirty-one (52%) underwent fine needle aspiration to confirm suspicious areas but only 27 (87%) of these provided adequate diagnostic material.

Surgical treatment involved local excision (35, 58%) or mastectomy - total in 19 (32%) and subcutaneous in six (10%).

Treatment policy at that time (H.D. Sinnett *et al.*, personal communication) was to recommend mastectomy for patients with 'extensive' intraduct disease; this was diagnosed preoperatively on mammography or after the initial biopsy had demonstrated extensive DCIS in the specimen. Mastectomy was also advised for those patients with associated Paget's disease of the nipple. All patients were given the choice of treatment after the potential risks of local recurrence following conservative surgery had been explained. Five of the six patients who chose subcutaneous mastectomy were under 45 years of age.

Representative H & E sections were reviewed by one of us (B.G). Margins of excision were assessed from the surgical and pathological reports at the time of operation and were judged to be complete in 53 (88%) cases.

Follow-up was available in all patients; (median 9 years, range 4-16 years). Follow-up included three monthly clinical examinations for the first two years and then at increasing intervals. Patients who had been treated by local excision also had yearly mammography. Aspiration cytology was not performed routinely unless suspicious areas were found clinically or radiographically.

Multivariate analysis was performed using Cox's regression in a stepwise fashion.

### Results

Patient clinical characteristics are shown in Table I. Histological subtypes of DCIS, papillary, cribriform, comedo or solid, were distributed fairly equally between the two groups but a number contained mixtures of more than one subtype.

The clinical signs of local recurrence were lump or nodularity in 17 cases and nipple change or discharge in five cases. Four cases (who were asymptomatic) were detected by mammography without associated clinical signs.

The diagnosis of local recurrence was made clinically in 16/26 cases, mammographically in 15/20 cases and by fine needle aspiration cytology in 10/15 cases. These are described in Table II.

A further five biopsies for suspected local recurrence were

Correspondence: P. Price, Department of Clinical Oncology, Royal Postgraduate Medical School, Hammersmith Hospital, Du Cane Road, London W12 OHS, UK.
Received 11 July 1989; and in revised form 2 January 1990.

**Table I** Patient characteristics

| | *Total mastectomy* | *Subcutaneous mastectomy* | *Local excision* |
|---|---|---|---|
| Median age | 54 | 37 | 50 |
| (range) | (31-78) | (29-55) | (33-67) |
| Clinical size[a] | | | |
| Lump <2 cm | 4 (21) | 2 | 5 (14) |
| >2 cm | 3 (16) | 0 | 2 (6) |
| Lump of unknown size/nodularity | 3 (16) | 2 | 11 (31) |
| No lump | 9 (47) | 2 | 17 (49) |
| Total | 19 | 6 | 35 |

[a]No significant differences between groups; figures in parentheses are %.

**Table II** Diagnosis of local recurrence in 26 women

| | *Number of patients with recurrence examined* | *Percentage positive* |
|---|---|---|
| Clinical examination | 26 | 62 |
| Mammography | 20 | 75 |
| Aspiration cytology | 15 | 67 |

*P*, non-significant.

carried out. Three patients had a lump or area of localised nodularity on clinical examination and two had suspicious microcalcification on their mammograms. However, the pathology in these five cases was benign.

Twenty-six patients have already recurred locally, 1/19 following total mastectomy, 3/6 after subcutaneous mastectomy and 22/35 with local excision. The 7-year recurrence-free percentages (95% confidence intervals) for the groups were total mastectomy 94% (84-100%), subcutanous mastectomy 44% (1-88%) and local excision 45% (28-55%) ($\chi^2$ trend = 11.9, $P<0.001$).

Of those who recurred following local excision alone, 19/22 (86%) did so at or near the original site of disease, while 2/22 (10%) had Paget's disease of the nipple as the presenting feature. Of the three patients who recurred locally after subcutaneous mastectomy, two developed Paget's disease of the nipple and one recurred in residual breast tissue at the edge of the prosthesis. The mastectomy patient recurred in the scar.

Of the 26 patients who relapsed locally, 14 did so with invasive disease (total mastectomy 1/1, subcutaneous mastectomy 1/3 and local excision 12/22). The 7-year invasive recurrence-free percentages (95% confidence intervals) for the groups were total mastectomy 94% (84-100%), subcutaneous mastectomy 75% (32-100%) and local excision 71% (56-87%) ($\chi^2$ trend = 4.17, $P<0.05$). Of the 12 patients who recurred with invasive disease in the local excision group, two have already developed metastases. A further two patients without local recurrence have developed distant metastases.

Figure 1 demonstrates the actuarial time to local recurrence in the breast. Local recurrence occurred at regular intervals throughout follow-up in each of the three treatment groups. Local recurrence with invasive disease similarly occurred steadily over time (Figure 2).

There were only seven deaths, three of which were attributed to causes other than cancer. Overall survival showed a trend favouring patients with local excision ($\chi^2$ trend = 3.45, $P<0.06$). Seven-year survival rates were 97%, 100% and 78% for the local excision, subcutaneous mastectomy and total mastectomy groups respectively.

There was no evidence of an improved recurrence free rate in 12 patients who were asymptomatic compared with those who were symptomatic at presentation ($\chi^2 = 0.63$, n.s.). There was no difference in recurrence free rates between patients presenting with a lump and nodularity and those presenting without ($\chi^2 = 0.05$, n.s.).

Treatment of the local recurrence depended on the original treatment of the primary tumour and whether invasive disease had developed (Table III). The local excision group

**Figure 1** Local recurrence by initial surgical treatment. ———, Local excision ($n = 35$); -----, subcutaneous mastectomy ($n = 6$); ——·——, total mastectomy ($n = 19$).

**Figure 2** Time to recurrence with invasive disease. ———, Local excision ($n = 35$); -----, subcutaneous mastectomy ($n = 6$); ——·——, total mastectomy ($n = 19$).

was usually treated conservatively again. Four of the eight of those treated by local excision for their first local recurrence have recurred a second time within the breast, all at or near the original site of disease. Two of these four have progressed to invasive disease at second recurrence.

Univariate and multivariate analysis were performed to determine predictors of local recurrence and invasive local recurrence. The only factor which correlated with both local and invasive recurrence on multivariate analysis was conservative surgery. The results are summarised in Table IV. Patients treated with conservative surgery are 4.71 times as likely to recur locally at any particular time during follow-up as those patients treated by mastectomy alone. Similarly, patients treated with conservative surgery are 4.05 times as likely to recur with locally invasive disease.

Factors which did not predict for local control on univariate and multivariate analysis included age, original size of tumour (patients without measurable tumours were considered to have a size of 0) and margins of excision.

**Table III** Treatment of local recurrence

| | *Initial treatment* | | |
|---|---|---|---|
| *Treatment of recurrence* | *Total mastectomy* | *Subcutaneous mastectomy* | *Local excision* |
| Local excision | – | 1 | 7 |
| Local excision + RT | 1 | – | 9 |
| Subcutaneous mastectomy | – | – | 1 |
| Total mastectomy | – | 1 | 5 |
| Radiotherapy alone | – | 1 | – |
| Total | 1 | 3 | 22 |

**Table IV** Predictors of local recurrence and local recurrence with invasive disease

| *Multivariate analysis* | *Hazard ratio (95%) CI)* | *P* |
|---|---|---|
| Local recurrence (overall): conservative surgery | 4.71 (1.59–14.0) | 0.001 |
| Local recurrence (invasive): conservative surgery | 4.05 (1.00–18.7) | 0.03 |

### Discussion

DCIS has traditionally been treated by mastectomy with the aim of cure. There are three main arguments in favour of this approach. First, the eradictation of multicentre foci of DCIS suspected to be present in the rest of the breast. Multicentric foci have been reported in 13–40% of breasts at subgross sectioning (Fisher *et al.*, 1975; Lagios, 1977; Lagios *et al.*, 1982; Schwartz, 1980). Second, the removal of occult invasive disease at foci in other breast quadrants. This is estimated as occurring in 6–21% of breasts after excision of DCIS (Brown *et al.*, 1976; Lagios *et al.*, 1982; Rosen *et al.*, 1979). Finally, mastectomy should prevent local recurrence or invasive disease arising from residual DCIS after local excision. The high incidence of this residue can be as high as 56% in mastectomy specimens following initial excision of DCIS (Rosen *et al.*, 1979).

Recently, a more conservative approach to treatment of pure DCIS has been advocated with the emergence of conservative management of early stage breast cancer and a reluctance to treat preinvasive disease more radically than invasive disease. With the introduction of national screening programmes the diagnosis of pure DCIS is expected to rise and with it the expectation of breast conserving surgery.

Local recurrence following local excision alone in this group of patients is 55% at 7 years. This compares with a local recurrence rate of 23% at 3 years and 15% at 4 years reported in two other series following excision alone for DCIS (Fisher *et al.*, 1986; Lagios *et al.*, 1982). Two cases recurred within 6 months of local excision and were thought to represent incompletely excised tumour. Recurrences have continued to appear throughout the follow-up period. While local recurrence within the breast is generally controllable, the concern is that cure has been jeopardised, as recurrence carries with it the risk of progression to invasive disease.

Local recurrence in this series carries a 54% risk of progression to invasive disease, which is similar to that reported in four series in which DCIS had been treated with breast conservation (Fisher *et al.*, 1986; Montague, 1984; Recht *et al.*, 1985; Zafrani *et al.*, 1986).

From our series it would appear difficult to predict clinically or pathologically those individual patients who are likely to recur or progress. Pathological categories defining precise extent of disease were not used as these could only be correctly designated if all the tumour was examined with multiple sections; this must be combined with an accurate assessment of the extent of the disease using subgross analysis in conjunction with adequate margin sampling. Tumour size did not predict for progression to invasive disease; but less than half of our cases presented with a palpable tumour. There was no difference in recurrence free rates between the group with or without tumour. This is contrary to suggestions by Lagios *et al.*, (1982) that the degree of multicentricity and occult invasion are related to tumour size.

In future selection of patients who are most likely to recur or progress may depend on other biological markers. For example, it has been suggested that tumour ploidy (Carpenter *et al.*, 1987) may be associated with an increased likelihood of recurrence. The over expression of the oncogene c-*erb*B-2 reported in 44% of evaluable DCIS cases presenting to this breast unit (Gusterson *et al.*, 1988) may provide a marker of biologically distinct local disease and is to be investigated further.

From our experience it would appear that local excision alone may be inadequate local treatment for patients presenting with DCIS. Furthermore, judging from the six patients in the group, subcutaneous mastectomy would appear to offer no advantage over a more conservative approach. In one of these patients diagnosis of local recurrence may have been delayed due to the tense capsule around the prosthesis. Mastectomy offers the best hope of local control for DCIS raising at least the probability of cure, but this still carries a small risk of local recurrence and metastatic disease. The former could come from residual foci after 'incomplete' mastectomy and the latter from undetected (micro) invasive foci.

Follow-up and survival data are not sufficient at present to enable a comparative statement to be made. Conclusions about optimal therapy which may be drawn from these pateints may not be applicable in the future when an increasing number of patients are diagnosed by screening mammography at either an earlier stage of disease or even with an entirely different biological disease.

Local excision followed by breast irradiation may provide an alternative approach for some patients, and results of the EORTC Protocol (10853) and NSABP (B17) prospective trials comparing local excision with local excision and radiotherapy for DCIS both presenting with symptoms and at screening are awaited.

In conclusion, until biological markers are found for those tumours most likely to recur or progress to invasive disease, conservative surgery for DCIS should only be considered if careful follow-up is available and the risks of local recurrence accepted. This work underlines the need for a national policy for disease management and clinical trials in screen-detected DCIS.

We would like to thank Dr J.R. Yarnold for his helpful discussions and Miss Jane Regan for her skilful secretarial assistance.

### References

ASHIKARI, R., HAJDU, S.I. & ROBBINS, G.F. (1971). Intraduct carcinoma of the breast (1960–1969). *Cancer*, **28**, 1182.

ASHIKARI, R., HUVOS, A.G. & SNYDER, R.E. (1977). Prospective study of non-infiltrating carcinoma of the breast. *Cancer*, **39**, 435.

BROWN, P.W., SILVERMAN, J. & OWENS, E. (1976). Intraductal 'non-infiltrating' carcinoma of the breast. *Arch. Surg.*, **111**, 1063.

CARPENTER, R., MATTHEWS, J., GIBBS, N., THOMAS, B., BOULTER, P. & COOKE, T. (1987). Prognostic value of cellular DNA content in the management of ductal carcinoma in situ of the female breast. *Br. J. Cancer*, **56**, 859.

FARROW, J.H. (1970). Current concepts in the detection and treatment of the earliest of the early breast cancers. *Cancer*, **25**, 468.

FISHER, E.R., GREGORIO, R., REDMOND, C., VELLIOS, F., SOMMERS, S.C. & FISHER, B. (1975). Pathological findings from the national surgical adjuvant breast project (protocol no. 4). I. Observations concerning the multicentricity of mammary cancer. *Cancer*, **35**, 247.

FISHER, E.R., SASS, R., FISHER, B., WICKERMAN, L., PAIK, S.M. AND COLLABORATING NSABP INVESTIGATORS (1986). Pathological findings of the national surgical adjuvant breast project (Protocol 6). I. Intraductal carcionoma (DCIS). *Cancer*, **57**, 197.

GUSTERSON, B.A., MACHIN, L.G., GULLICK, W.J. & 5 others (1988). Immunohistochemical distribution of c-erB-2 in infiltrating and in-situ breast carcinoma. *Int. J. Cancer*, **42**, 842.

LAGIOS, M.D. (1977). Multicentricity of breast carcinoma demonstrated by routine correlated serial subgross and radiographic examination. *Cancer*, **40**, 1726.

LAGIOS, M.D., WESTDAHL, P.R., MARGOLIN, F.R. & ROSE, M.R. (1982). Duct carcinoma in situ. Relationship of extent of non invasive disease to the frequency of occult invasion, multicentricity, lymph node metastases and short term treatment failures. *Cancer*, **50**, 1309.

MONTAGUE, E.D. (1984). Conservative surgery and radiation therapy in the treatment of operable breast cancer. *Cancer*, **53**, suppl. 3, 700.

RECHT, A., DANNOFF, B.S., SOLIN, L.J. *et al.* (1985). Intraductal carcinoma of the breast; results of treatment with excisional biopsy and irradiation. *J. Clin. Oncol.*, **3**, 1339.

ROSEN, P.P., SENIE, R., SCHOTTENFELD, D. & ASHIKARI, R. (1979). Non-invasive breast carcinoma. Frequency of unsuspected invasion and implications for treatment. *Ann. Surg.*, **189**, 377.

SCHWARTZ, G.P., PATCHEFSKY, A.S., FEIG, S.A., SHABER, G.S. & SCHWARTZ, A.B. (1980). Multicentricity of non-palpable breast cancer. *Cancer*, **45**, 2913.

SUNSHINE, J.A., MOSELY, H.S., FLETCHER, W.S. & KRIPPAEHNE, W.W. (1985). Breast carcinoma in situ: a retrospective review of 112 cases with a minimum 10 year follow up. *Am. J. Surg.*, **150**, 44.

WESTBROOK, K.C. & GALLAGER, H.S. (1975). Intraduct carcinoma of the breast: a comparative study. *Am. J. Surg*, **130**, 667.

ZÂFRANI, B., FOURQUET, A., VILCOQ, J.R., LEGAL, M. & CALLE, R. (1986). Conservative management of intraductal breast carcinoma with tumourectomy and radiation therapy. *Cancer*, **57**, 1299.